# PatchProt: hydrophobic patch prediction using protein foundation models

**DOI:** 10.1093/bioadv/vbae154

**Published:** 2024-10-14

**Authors:** Dea Gogishvili, Emmanuel Minois-Genin, Jan van Eck, Sanne Abeln

**Affiliations:** Bioinformatics, Computer Science Department, Vrije Universiteit Amsterdam, Amsterdam, 1081 HV, The Netherlands; AI Technology for Life, Department of Computing and Information Sciences, Department of Biology, Utrecht University, Utrecht, 3584 CS, The Netherlands; Bioinformatics, Computer Science Department, Vrije Universiteit Amsterdam, Amsterdam, 1081 HV, The Netherlands; AI Technology for Life, Department of Computing and Information Sciences, Department of Biology, Utrecht University, Utrecht, 3584 CS, The Netherlands; Bioinformatics, Computer Science Department, Vrije Universiteit Amsterdam, Amsterdam, 1081 HV, The Netherlands; AI Technology for Life, Department of Computing and Information Sciences, Department of Biology, Utrecht University, Utrecht, 3584 CS, The Netherlands

## Abstract

**Motivation:**

Hydrophobic patches on protein surfaces play important functional roles in protein–protein and protein-ligand interactions. Large hydrophobic surfaces are also involved in the progression of aggregation diseases. Predicting exposed hydrophobic patches from a protein sequence has shown to be a difficult task. Fine-tuning foundation models allows for adapting a model to the specific nuances of a new task using a much smaller dataset. Additionally, multitask deep learning offers a promising solution for addressing data gaps, simultaneously outperforming single-task methods.

**Results:**

In this study, we harnessed a recently released leading large language model Evolutionary Scale Models (ESM-2). Efficient fine-tuning of ESM-2 was achieved by leveraging a recently developed parameter-efficient fine-tuning method. This approach enabled comprehensive training of model layers without excessive parameters and without the need to include a computationally expensive multiple sequence analysis. We explored several related tasks, at local (residue) and global (protein) levels, to improve the representation of the model. As a result, our model, PatchProt, cannot only predict hydrophobic patch areas but also outperforms existing methods at predicting primary tasks, including secondary structure and surface accessibility predictions. Importantly, our analysis shows that including related local tasks can improve predictions on more difficult global tasks. This research sets a new standard for sequence-based protein property prediction and highlights the remarkable potential of fine-tuning foundation models enriching the model representation by training over related tasks.

**Availability and implementation:**

https://github.com/Deagogishvili/chapter-multi-task

## 1 Introduction

Predicting large hydrophobic patches on the protein surfaces is a complex learning task (van Gils *et al.* 2022). Proteins typically hide hydrophobic residues within their core to avoid interaction with water, a phenomenon known as the hydrophobic effect (Dill 1985, 1990). When such *sticky* residues appear on the surface, they can play key roles in functional protein–protein, -ligand, or -membrane interactions (Chothia and Janin 1975, Young *et al.* 1994, Gowder *et al.* 2014), as well as induce amyloid fibril formation in the context of aggregation diseases (Chiti and Dobson 2006, Tuttle *et al.* 2016, Iadanza *et al.* 2018). Keeping these residues internal is thought to be a key strategy to avert protein aggregation (Dobson 2003, Abeln and Frenkel 2008, 2011). Hydrophobic areas on the surface of the protein can influence experimental processes, such as gel formation, protein crystallization (Wright and Dyson 1999), and separation techniques (Moruz and Käll 2017). Previously we developed a method to define the largest hydrophobic patch (LHP)—the largest connected hydrophobic area on the protein surface (van Gils *et al.* 2022). Additionally, we demonstrated the significance of exposed hydrophobic surfaces in terms of human disease (van Gils *et al.* 2022). LHPs can be used to identify aggregation-prone regions (Sankar *et al.* 2018) which pose significant hurdles for the development of therapeutic proteins, such as monoclonal antibodies (Redington *et al.* 2017, Sankar *et al.* 2018). Importantly, predicting the exposure of hydrophobic residues on the protein surface is not a trivial problem. Traditional methods predict the majority of hydrophobic residues to be fully buried (Kyte and Doolittle 1982, van Gils *et al.* 2022). The continued evolution of the tools and methodologies is needed to deepen our understanding of protein hydrophobicity, especially in the context of neurodegenerative diseases.

The ability to predict structural and functional protein properties directly from a primary sequence is of paramount importance for unravelling its function in the absence of experimental structural information or predictions of low confidence. Various computational tools mostly focus on either local (per residue) or global (protein level) predictions, by taking a protein sequence as input and outputting a value or class per amino acid or protein chain (Hou *et al.* 2022). Typical local tasks are the prediction of secondary structural elements, backbone geometry, post-translational modifications, and residues on protein–protein interfaces (Klausen *et al.* 2019, Capel *et al.* 2022). Properties, such as cellular localization, expression levels, and functional annotations are mostly predicted at the global level (Almagro Armenteros *et al.* 2017, Waury *et al.* 2024). Due to the lack of local annotations for training, many prediction methods focus on tasks at the global level. Nevertheless, these global prediction tasks form a class of hard prediction problems, including solubility, aggregation propensity, stability, turnover, and LHPs (Khurana *et al.* 2018, van Gils *et al.* 2022, Housmans *et al.* 2023). While local values can typically be summarized as global values, the reverse process is not possible.

Multitask deep learning architectures were previously shown useful to enrich a model representation, where there is a scarcity of annotated data for the task of interest (Capel *et al.* 2022). Fine-tuning foundation models, which have been pretrained on a vast amount of data, allows for effectively adapting a model to a new task even with limited datasets (Høie *et al.* 2022, Perez *et al.* 2023).

Machine learning has long leveraged evolutionary profiles in multiple sequence alignments (Rost *et al.* 1994), to predict local or global protein features, including three-dimensional (3D) structure (Camacho *et al.* 2009, Klausen *et al.* 2019, Jumper *et al.* 2021). Generating a multiple sequence alignment is typically a rate-limiting step as it involves an exhaustive search of homologs (Camacho *et al.* 2009, Remmert *et al.* 2011, Potter *et al.* 2018, Mirdita *et al.* 2019). Since the development of transformer-based models (Vaswani *et al.* 2017) large language models have revolutionized the field of natural language processing (Chowdhary 2020) and have been successfully applied to the analysis of protein sequences (Elnaggar *et al.* 2021). The information in a multiple sequence alignment can now partially be captured by a protein language model leading to an order-of-magnitude acceleration of high-resolution structure prediction (Heinzinger *et al.* 2019). Evolutionary Scale Models (ESM) developed by Meta were trained on predicting masked residues in protein sequences and have recently presented promising results in protein folding prediction (Lin *et al.* 2023).

This study builds upon protein foundation models and draws inspiration from recent advancements in deep learning architectures (Høie *et al.* 2022). Current methodologies in protein property predictions focus on either global or local predictions. Here, we aimed to bridge this gap in current technology. The novelty of this framework lies in several key aspects. First, we introduce a multi-task learning approach that simultaneously predicts both global and local (L)HP values, a feature that has not been previously explored at the residue level. This dual-focus methodology enables the model to learn commonalities and differences across tasks to improve generalization, allowing us to explore other (un)related global tasks with limited data availability. Hence, we extended our train and test datasets with normalized expression annotations. This addition was inspired by our previous study, where we showed that highly hydrophobic proteins are generally expressed at lower levels in the human proteome (van Gils *et al.* 2022). Second, our parameter-efficient fine-tuning methodology enabled us to effectively train large transformer models, overcoming one of the major bottlenecks of large language models. Our framework allowed us to (i) outperform the state-of-the-art methods in primary tasks; (ii) improve the global LHP predictions; (iii) obtain the first model that can predict (L)HPs on a residue level. Moreover, PatchProt demonstrated the possibility of foundation models and multitask strategies to improve the accuracy of protein property predictions even with sparse datasets.

## 2 Methods

### 2.1 Standard dataset

To benchmark the performance of PatchProt for the primary prediction tasks, training and test datasets were obtained from previous work and used to develop NetSurfP-2 and -3 (Klausen *et al.* 2019). The curated training dataset contains 10 848 proteins retrieved from PDB with a sequence similarity ≤25%. For a fair comparison, our models were evaluated on fixed test sets, and the performance values for the NetsurfP models are shown as reported in the latest publication (Høie *et al.* 2022). Test datasets CASP12 (*n* = 21), CB513 (*n* = 513) and TS115 (*n* = 115) are classic datasets for evaluating protein feature prediction models. All residues in each chain in the training dataset are annotated by an eight-state secondary structure (Q8), three-state secondary structure (Q3), relative solvent-accessible (RSA) area, absolute solvent-accessible (ASA) area, and ϕ and ψ dihedral angles with the DSSP software. Residues present in the chain RefSeq sequence, but not in the solved structure, were defined as disordered (Dis) (Klausen *et al.* 2019, Høie *et al.* 2022). As stated by Høie *et al.* and Klausen *et al.*, no atomic coordinates are available for residues labelled as disordered and thus such residues could not be annotated with other features (Klausen *et al.* 2019, Høie *et al.* 2022).

### 2.2 Dataset expansion

To investigate auxiliary tasks, we extended the datasets described above with more features, including (L)HP area, normalized RNA expression, and species, ultimately combining residue-based and global protein properties (Table 1). For LHP annotations, we utilized the MolPatch method to calculate the area of hydrophobic patches based on the 3D structure of all protein chains (van Gils *et al.* 2022). MolPatch is a structure-based tool which creates a point cloud on the solvent-excluded protein surface. It retains the edges between the node pairs labelled as hydrophobic, extracts individual network components and calculates accessible surface area, providing the rank and the size of hydrophobic patches in a given PDB structure of a protein. The output file indicates a residue type and number included in (L)HPs across the sequence (van Gils *et al.* 2022). In this work, the structure-based MolPatch definitions for the (largest) hydrophobic patches are considered the ground truth. The relevance of these definitions was shown by hydrophobic measures and by assessing the functional role of the LHPs in previous work (van Gils *et al.* 2022). To calculate hydrophobic patches, PDB structures in the existing datasets were retrieved. During the dataset expansion, amino acid sequences of PDB structures were compared with the sequences of the NetSurfP dataset and entries with a sequence match of more than 95% amino acids were selected and annotated (in total, 10 594 chains). The (L)HP global (g) indicates the size of the (largest) hydrophobic patch for the whole chain, while (L)HP local (l) depicts a binary annotation per amino acid, whether or not a specific residue is in the (largest) hydrophobic patch. Previously, we have shown that only lowly expressed human proteins (based on mRNA expression), are predicted to have large hydrophobic patches (van Gils *et al.* 2022). Here, we explore whether adding normalized expression values would aid LHP predictions. For normalized expression annotations, RNA consensus tissue gene data were obtained from the human protein atlas (Uhlén *et al.* 2015) as described in the recent study (van Gils *et al.* 2022). To obtain a single expression value for every gene, the highest expression value was selected among all the tissues in which each gene was expressed. To obtain distinct groups, obtained values were grouped and the two lowest and the two highest deciles were selected for the prediction task (in total 618 chains). Additionally, we assigned labels to proteins based on the ten most common species including it as a prediction task. These labels encompass the ten most prevalent species, including: *H. sapiens* (*n* = 1638), *E. coli* (*n* = 615), *S. cerevisiae* (*n* = 393), *M. musculus* (*n* = 291), *B. subtilis* (*n* = 153), *M. tuberculosis* (*n* = 144), *P. aeruginosa* (*n* = 143), *T. thermophilus* (*n* = 135), *A. thaliana* (*n* = 133), and *T. maritima* (*n* = 122). Importantly, the added annotations do not completely cover our training and test datasets. To handle missing values, we ignored the loss value of the missing annotations in the multitask loss (see the multitask loss in Section 2).

**Table 1.** Training and test data with additional features.

Dataset	Task	Feature	Source	Train	Test	Test	Test
				HHBlits	CASP12	CB513	TS115
Original (Klausen *et al.* 2019, Høie *et al.* 2022)	Local	Q8, Q3, RSA, ASA	DSSP	10 848	21	513	115
		ϕ , ψ, Disorder					
Added	Global	TASA, THSA	DSSP	10 848	21	513	115
Added	Global, Local	(L)HP	MolPatch	9991	20	470	113
Added	Global	NX	HPA	579	1	31	7
Added	Global	SP	RCSB	3528	4	181	115

Q8, eight-state secondary structure; Q3, three-state secondary structure; RSA, relative solvent-accessible area; ASA, absolute solvent-accessible area; TASA, total accessible surface area; THSA, total hydrophobic surface area; (L)HP, (largest) hydrophobic patch; NX, normalized expression; SP, species.

### 2.3 Model

**Figure 1.**
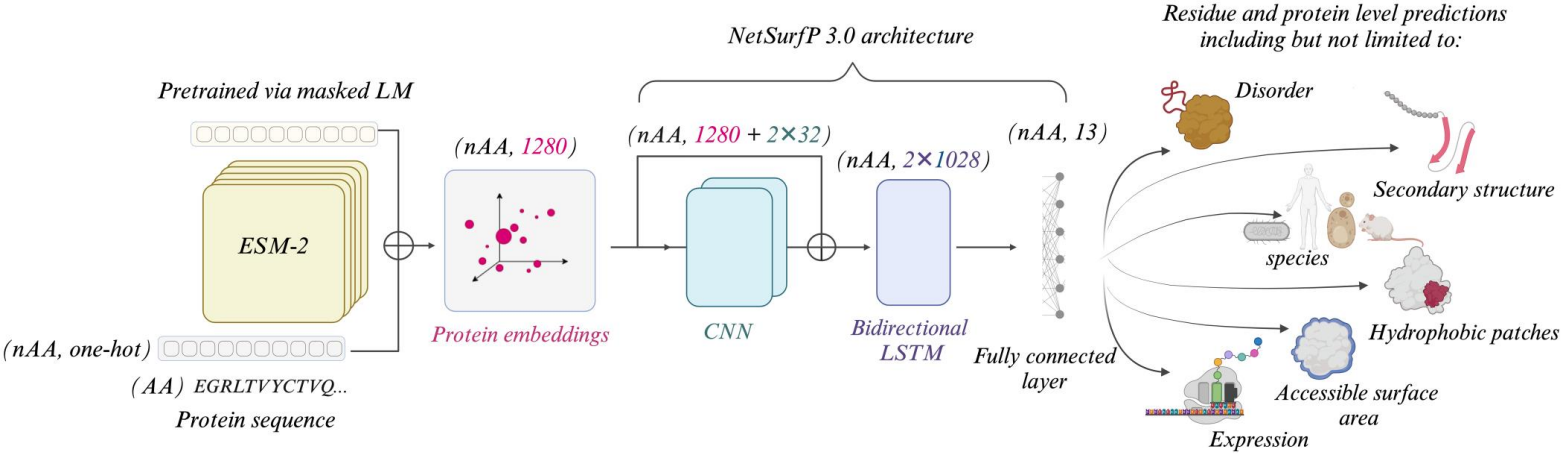
Model architecture. The model takes protein sequence as input and predicts both global and local protein properties. The model consists of an embedding output from ESM-2 protein language model (Lin *et al.* 2023) and the downstream architecture similar to NetSurfP-3 (Høie *et al.* 2022). Additionally, a parameter-efficient fine-tuning strategy was implemented (Fig. S1) (Hu *et al.* 2021, Pfeiffer *et al.* 2021). The decoding head consists of a residual block with two CNN layers and a two-layer bidirectional long short-term memory (BiLSTM) network. The output is fed into a fully connected layer to provide predictions for all residues- and protein-level tasks.

Our approach to building a deep learning model architecture was inspired by NetSurfP-3 (Høie *et al.* 2022). In addition, we implemented an efficient fine-tuning strategy and explored a wide range of related global and local tasks. Figure 1 shows the overview of the model architecture. We utilized the embedding output from the ESM-2 protein language model (Lin *et al.* 2023) and applied the downstream architecture to obtain predictions.

### 2.4 Architecture, input and output

To predict properties for a given protein, our input shape is defined as (nAA,OH), where *nAA* represents the number of amino acids in the protein and *OH* signifies the one-hot-encoded amino acid at the respective index (OH=20). For a given batch size, the input would be (P,nAA,OH), where *P* denotes the number of proteins in a single batch. PatchProt, utilizing ESM2, initially projects this (nAA,OH) matrix into the embedding space, resulting in a matrix of shape (nAA,H), with *H* representing the embedding size (in our case, 1280).

Our decoding head resembles the NetSurfP-3 architecture as described in Høie *et al.* (2022). It features two separate CNN layers passed to a two-layer bidirectional Long Short-Term Memory (LSTM) network. The output is fed into a fully connected layer providing predictions for all residue and protein-level tasks. This design aims to extract information from the embeddings generated by the ESM-2 model, thus enhancing our model’s capacity to utilize the representations offered by protein language models (Fig. 1). Consequently, post-embedding extraction, we apply a 1D-CNN to incorporate additional features to each residue, resulting in a matrix of shape $\left(\mathrm{nAA},H+O_{\text{CNN}}\times N_{\text{CNN}}\right)$, where $O_{\text{CNN}}$ represents the number of CNN output channels (we assume uniformity across all CNN outputs, as is the case in PatchProt, where we use 2 CNNs with the output size of 32), and $N_{\text{CNN}}$ denotes the number of CNNs applied. To further enhance feature extraction, we apply bidirectional LSTM to these embeddings, resulting in a matrix of size $\left(\mathrm{nAA},2\times H_{\text{LSTM}}\right)$, with $H_{\text{LSTM}}$ representing the hidden size of the LSTM (multiplied by 2 due to the bidirectional nature). Concatenating the forward and backward representations (where $H_{\text{LSTM}}=1024$ in PatchProt), we proceed to employ a linear layer for predicting the properties of each amino acid. Given that our multitask model predicts multiple classes and values on both protein and residue levels simultaneously, the output is of shape (nAA,nTasks). For all 13 prediction tasks, each has its dimensions. For instance, if classification among 8 different classes is required, the output size would be 8.

### 2.5 Combining global and local tasks

Notably, our datasets contain additional global features. Hence, we developed a model which can predict both global features (LHP, species, and expression) and local features [(L)HP, ASA, RSA, SS and disorder]. To achieve this, for the global tasks, we simply sum the prediction of each amino acid for the specific task and use this as a prediction:

(1) $$Y=\sum_{i}^{N}y_{i}$$

The result is of shape (1) since we predict one value per protein. This approach has one key advantage as it can emphasize the impact of each residue on the final label of the respective protein. This decomposition of the global features on a residue basis allows for an easy interpretation of our results.

### 2.6 Multitask loss

Multitask learning is a powerful approach in machine learning where a model is trained on multiple tasks simultaneously, leveraging commonalities and differences across tasks to obtain robust representations and improve generalization (Kendall *et al.* 2018, Liu *et al.* 2019). However, one of the key challenges in multitask learning is effectively balancing the learning across tasks, as each task may have different levels of difficulty and importance. This necessitates the development of strategies to dynamically adjust the emphasis on each task during the training process.

The uncertainty-based loss, described by Liebel *et al.*, has shown promise in dynamically balancing the contribution of different tasks based on their levels of uncertainty by weighting each with a factor $\sigma_{t}$ (Liebel and Körner 2018). To calculate individual losses, mean squared loss [RSA, ϕ, ψ, total accessible surface area (TASA), total hydrophobic surface area (THSA), global LHP] and cross-entropy loss [Q8, Q3, disorder, local (L)HP, species, expression] are used.

#### 2.6.1 Scaling losses according to uncertainty

When dealing with a multi-task learning scenario, where we aim to predict multiple properties using a single model, it is crucial to design a loss function that adequately accounts for the differences in tasks. These differences can arise from variations in scales or units, or even from the nature of tasks, such as classification or regression.

In the case of a regression model, we typically assume that the true values are normally distributed around their predicted counterparts (Kendall *et al.* 2018): $y_{n}∼N(f(x_{n}),\sigma^{2})$. Where $\sigma^{2}$ represents the aleatoric uncertainty in our data (which is not reducible) and $f(x_{n})$ denotes the model’s prediction given the *n*th input from all inputs in *x*. We assume in our case that the uncertainty does not depend on the data (homoscedastic). During model optimization, we would like to maximize the likelihood p(y|x). This likelihood can be written as:

(2) $$\Pi_{n}^{N}p(y_{n}|f(x_{n}))$$

and, respectively as:

(3) $$\Pi_{n}^{N}\frac{1}{\sigma \sqrt{2\pi }}{\;\text{exp}\;}^{\left(-\frac{1}{2}{\left(\frac{y_{n}-f(x_{n})}{\sigma }\right)}^{2}\right)}$$

where *N* represents the total number of inputs. Typically, we take the log and multiply by −1 to minimize (easier to handle numerically). Additionally, we get rid of the constant, which brings us to the loss function (Kendall *et al.* 2018, Liebel and Körner 2018):

(4) $$\frac{1}{2\sigma^{2}}L+\text{log}(\sigma )$$

where:

(5) $$L=||y,f(x)|{|}^{2}=\sum_{n}^{N}(y_{n}-f(x_{n}){)}^{2}$$

While in a single-task model, we remove the uncertainty term σ (as we consider it a constant), in multitask modelling we use the uncertainty to weigh different tasks. If we have two regression tasks with losses $L_{1}$ and $L_{2}$ similar to the one we computed above, but with different uncertainty $\sigma_{1}$ and $\sigma_{2}$ because they are not on the same scale/unit or simply because one of the tasks is noisier. We usually assume that the two tasks are independent:

(6) $$p(y_{1},y_{2}|f(x),f(x))=p(y_{1}|f(x))p(y_{2}|f(x))$$

(7) $$L_{1,2}=\frac{1}{2\sigma_{1}^{2}}L_{1}+\frac{1}{2\sigma_{2}^{2}}L_{2}+\text{log}(\sigma_{1}\sigma_{2})$$

where $\text{log}(\sigma_{1}\sigma_{2})$ is a regularization term that prevents the uncertainty from increasing and masks one of the two tasks. This approach can also be used for classification tasks (where σ represents the temperature, analogue of the uncertainty for categorical distribution). The multitask loss function used in this paper is derived from (7):

(8) $$L_{\text{multi}}=\sum_{t\in \tau }\frac{L_{t}}{2\sigma_{t}^{2}}+\ln(1+\sigma_{t}^{2}),$$

where τ represents the set of tasks, $L_{t}$ is the loss function for task *t* (mean squared loss and cross-entropy loss), and $\sigma_{t}$ is the uncertainty term for task *t*. To prevent negative loss values $\ln(1+\sigma_{t}^{2})$ is administered in the approach instead of $\text{log}(\sigma_{t})$ (Liebel and Körner 2018).

The summation of these components across all tasks τ, where τ includes only the tasks with non-null values in a given batch, forms the final loss function. This formulation allows the model to prioritize tasks based on their current level of uncertainty, potentially leading to more effective and efficient learning.

During optimization, one way to implement this approach is to learn the uncertainty parameters σ during training (as we cannot infer them before training) and to calculate the loss for each batch and adjust the uncertainty weights according to the optimization objective (Lin *et al.* 2021).

### 2.7 Fine-tuning strategy

To efficiently fine-tune the foundation model, we adopted recent advancements in parameter-efficient fine-tuning known as Low-Rank Adaptation (LoRA; Hu *et al.* 2021) (see online supplementary material for a colour version of this figure, Fig. S1). With an expansion of large language models, conventional methods of fine-tuning have grown impractical. LoRA has been demonstrated to significantly reduce the computational cost without sacrificing performance by freezing pre-trained model weights and introducing trainable rank decomposition matrices (Hu *et al.* 2021).

The underlying principle of LoRA is based on the hypothesis that the change of weights during the fine-tuning process has an intrinsically low rank. This suggests that, rather than updating an entire weight matrix in each dense layer, only a few parameters are adjusted. Essentially, this hypothesis implies that most of the columns of the weight matrix are linearly dependent, eliminating the need to individually adjust each column. By restricting the extent of the model’s changes during fine-tuning, LoRA provides a form of implicit regularization, which is particularly useful for limited datasets. LoRA effectively constrains the learning process by focusing on important features and reduces the risk of overfitting by only updating small, low-rank matrices instead of all the parameters (Hu *et al.* 2021).

In LoRA, $W_{0}$ denotes the original weight matrix of a specific layer within a pre-trained model, while Δ*W* represents adjustments to weight changes to improve the layer’s performance for new tasks. The final weights are obtained by adding $W_{0}$ and Δ*W* matrices. The principal innovation of LoRA lies in decomposing the weight change matrix Δ*W* into two lower-rank matrices, *A* and *B*, with dimensions r×d and d×r respectively:

(9) $$A\in R^{r\times d},\;B\in R^{d\times r},\;r\ll d,$$

This approach significantly reduces the number of updated parameters (to 2*rd* from the layer’s original $d^{2}$), thereby enhancing the efficiency of the fine-tuning process. These updates are applied through residual connections, allowing us to modify the model’s behaviour with minimal changes to its pre-trained weights. The adapted output *h* for a new input *x* is computed as follows:

(10) $$h=W_{0}x+\Delta Wx=W_{0}x+\mathrm{BAx},$$

Here, ΔW=BA represents the weight adjustments through the low-rank decomposition, where only matrices *A* and *B* are updated, improving the efficiency of the training while maintaining the integrity of the pretrained model.

In our approach, we applied LoRA to every linear layer within the original transformer architecture (Vaswani *et al.* 2017), targeting not only queries, keys and values matrices but also the projection layer in the multi-head attention and the feed-forward network in the transformer as shown in online supplementary material for a colour version of this figure, Fig. S1. Notably, when using LoRA for efficient fine-tuning, the multi-task loss is applied to the whole architecture (Fig. 1) (in this case, LoRA introduces low-rank matrices that are trainable and are added to the pre-existing weights of the ESM-2 model). If there is no fine-tuning chosen, then the multi-task loss is applied to the CNN-LSTM part of the model following the generation of embeddings (as depicted in Fig. 1).

### 2.8 Handling long sequences

It is computationally expensive to generate embeddings for long FASTA sequences. Here we propose an approach to parse long sequences, by introducing a new parameter to achieve a better representation of long inputs. As in NetSurfP-3 (Høie *et al.* 2022), we divide the input into several parts of equal lengths of 1048 amino acids. Afterwards, instead of truncating only the end of the previous part, we truncate both embeddings and assemble the results.

### 2.9 Batch size correction

Combining global and local tasks is challenging in terms of batch sizes. Since every amino acid residue is a training sample for local features the model requires way fewer sequences to be trained compared to the global features, when it has to predict a single value per protein. Furthermore, as computing and storing the embeddings of large proteins is computationally heavy, a single GPU can only be used for a smaller batch size. To ensure that we learn the global features adequately, we need to maximize the batch size. For this, we applied gradient optimization techniques to allow a greater batch size on a single GPU, namely, gradient accumulation and gradient checkpointing. Using these techniques, we can increase our batch size of 2 (2 proteins) to 18. Using gradient checkpointing on half of the transformers allows us to increase the batch size to 3 and gradient accumulation can be used to increase the batch size (we accumulate the gradient over 6 batches) resulting in a virtual batch size of 6×3=18 molecules per batch.

### 2.10 Evaluating global largest hydrophobic patch predictions 

To benchmark global (protein level) predictions for the LHPs, we used the same test set of monomeric proteins as described previously (van Gils *et al.* 2022). We checked the overlap with the training dataset and removed all proteins with the matching PDB identifiers. The final test dataset for the global LHP predictions consisted of 346 monomeric proteins.

To assess the performance of PatchProt on global predictions for LHP values, we benchmarked our predictions against previously trained and reported models (van Gils *et al.* 2022), including the three-feature model (TFM) trained using a cubist regression in the CARET module, which uses the sequence length, number of hydrophobic amino acids and number of hydrophilic amino acids as input features (Kuhn 2008); The global feature model was trained on 31 global features using an XGBoost regressor (Chen and Guestrin 2016). Input protein-level features include amino acid count (20), hydrophobic and polar amino acid counts, sequence length, entropy, molecular weight, aromaticity, instability index, gravy score (average hydrophobicity), buried, isoelectric point and molar extinction coefficient. The LHP cannot be calculated from NetSurfP-2 predictions directly (Klausen *et al.* 2019, van Gils *et al.* 2022). Therefore, the NetSurfP-2-based model (NBM) is a random forest model trained on the total and relative hydrophobic surface area (THSA and RHSA) values predicted by NetSurfP-2. To predict the LHP from a sequence, the LHP calculated by MolPatch was used as a gold standard.

## 3 Results

In this study, we first aimed to explore the potential of foundation models and efficient fine-tuning strategies to improve primary protein property prediction tasks. Second, using multi-task learning, we set to obtain a well-performing (L)HP predictor on both residue and protein levels. Finally, we expanded the model and added auxiliary tasks with scarce annotations to ascertain the possibility of using limited datasets to take advantage of shared representations.

### 3.1 Improved secondary structure predictions

Before addressing the (L)HP prediction challenge, our goal was to develop an optimal architecture. We began by evaluating several models for predicting protein structural features and benchmarking them against state-of-the-art methods NetSurfP-2 and NetSurfP-3. Table 2 outlines the models and prediction tasks we examined. NetSurfP-2 is a state-of-the-art tool for protein secondary structure, solvent accessibility, and disorder from its primary sequence (Klausen *et al.* 2019). The replacement of time-consuming multiple sequence alignments with the ESM-1b protein language model (Rives *et al.* 2021) significantly decreased the runtime without compromising prediction accuracy (version 3.0) (Høie *et al.* 2022). ESM-2 represents META’s latest protein language model at the time of conducting this study (Lin *et al.* 2023). Our model (PatchProt) architecture integrates ESM-2 with a ResNet encoder and bidirectional LSTM head (similar to NetSurfP-3). PatchProt (SSE) was trained and tested on secondary structural features, similar to NetSurfP-2, NetSurfP-3, and ESM-2. In contrast, PatchProt (All) was trained and tested on secondary structural elements as well as auxiliary tasks, including (L)HP, species classification, and normalized expression values. Importantly, in each batch, the final multi-task loss function only considers tasks with non-null values. Additionally, we tested the impact of incorporating an efficient fine-tuning strategy (LoRA) on ESM-2, PatchProt (SSE), and PatchProt (All). Table 2 shows the comparison of different models on typical secondary structure component predictions across standard test datasets. ESM-2 alone achieves similar performance as NetSurfP-3 and outperforms it in almost all tasks (Table 2). PatchProt (All) with LoRA and auxiliary tasks leads to improved predictions in the majority of the SSE tasks and metrics (Table 2).

**Table 2.** Model performance when applying ESM-2 embeddings to predict protein local structure.

Test	LoRA	Model (Tasks)	RSA ↑	ASA ↑	Q8 ↑	Q3 ↑	Dis ↑	Dis ↓	Phi ↓	Psi ↓
dataset			(PCC)	(PCC)	(ACC)	(ACC)	(MCC)	(FNR)	(MAE)	(MAE)
CASP12	**-**	NetSurfP-2 (SSE)	0.728	0.739	**0.699**	0.810	0.653	0.015	20.90	32.80
	**-**	NetSurfP-3 (SSE)	0.707	0.722	0.669	0.791	0.621	0.024	21.25	33.92
	**-**	ESM-2 (SSE)	0.710	0.717	0.653	0.785	0.543	**0.013**	21.49	33.48
	√	ESM-2 (SSE)	0.707	0.724	0.666	0.777	0.559	0.021	20.60	32.50
	**-**	PatchProt (SSE)	**0.740**	**0.748**	0.695	**0.817**	**0.658**	0.026	**20.20**	**30.95**
	√	PatchProt (SSE)	0.720	0.735	0.683	0.792	0.579	0.029	20.39	31.78
	**-**	PatchProt (All)	0.730	0.738	0.667	0.799	0.583	0.024	20.80	31.33
	√	PatchProt (All)	0.724	0.741	0.685	0.795	0.592	0.032	20.42	32.42
CB513	**-**	NetSurfP-2 (SSE)	0.791	0.804	0.713	0.845	–	–	20.35	29.04
	**-**	NetSurfP-3 (SSE)	0.793	0.810	0.711	0.846	–	–	20.22	29.25
	**-**	ESM-2 (SSE)	0.791	0.804	0.682	0.836	–	–	20.76	30.28
	√	ESM-2 (SSE)	0.803	0.817	0.724	0.859	–	–	19.34	26.60
	**-**	PatchProt (SSE)	0.811	0.823	0.724	0.860	–	–	19.47	26.73
	√	PatchProt (SSE)	**0.816**	**0.828**	0.737	**0.868**	–	–	18.93	**25.56**
	**-**	PatchProt (All)	0.809	0.821	0.704	0.855	–	–	19.93	27.87
	√	PatchProt (All)	**0.816**	**0.828**	**0.738**	**0.868**	–	–	**18.83**	25.72
TS115	**-**	NetSurfP-2 (SSE)	0.771	0.793	0.740	0.849	0.624	**0.013**	17.40	26.80
	**-**	NetSurfP-3 (SSE)	0.776	0.799	0.749	0.856	0.662	0.015	17.16	25.80
	**-**	ESM-2 (SSE)	0.772	0.790	0.719	0.844	0.605	**0.013**	17.76	27.00
	√	ESM-2 (SSE)	0.785	0.805	0.753	0.858	0.646	0.016	16.76	24.42
	**-**	PatchProt (SSE)	0.796	0.812	0.757	0.867	**0.667**	0.016	16.67	23.75
	√	PatchProt (SSE)	0.794	0.813	0.763	0.869	0.650	0.014	16.37	**23.59**
	**-**	PatchProt (All)	0.792	0.809	0.739	0.861	0.650	0.014	17.03	24.83
	√	PatchProt (All)	**0.799**	**0.817**	**0.765**	**0.871**	0.649	0.016	**16.24**	23.67

Comparison of NetSurfP-2, NetSurfP-3, and our model—PatchProt on the CB513, TS115 and CASP12 datasets. Performance values for the NetsurfP models are reported as stated in the latest publication (Høie *et al.* 2022). ESM-2 consists of the ESM-2 embedding model with linear layers at its end for making predictions. SSE—secondary structure element [the model was only trained on basic secondary structure component tasks, eight- (Q8) and three-state secondary structure (Q3), RSA, ASA, and dihedral angles (ϕ, ψ)]. All indicates SSE + auxiliary tasks additionally include global tasks (TASA, THSA), (largest) hydrophobic patches (both global and local), species, and expression. Dis, disorder. √ indicates fine-tuning of ESM-2 using LoRA, while the rest of the model is fully trained. When the LoRA column displays a “–”, it means the ESM-2 model is entirely frozen, with only the head being trained. Each column reports an output variable with the same corresponding metrics reported in the previous study (Høie *et al.* 2022) for benchmarking purposes: Pearson correlation coefficient (PCC), accuracy (ACC), Matthews correlation coefficient (MCC), false negative rate (FNR), and MAE. Up- and down-facing arrows indicate metrics for which an improvement represents larger or lower values. For each dataset and prediction task, the values corresponding to the best performance are shown in bold.

### 3.2 Improved large hydrophobic patch predictions

It has been previously shown that predicting the global LHP area for a protein is not a trivial task (van Gils *et al.* 2022). By training an XGBoost regressor (Chen and Guestrin 2016) on basic protein characteristics, such as sequence length, and number of hydrophobic and hydrophilic residues, the performance of the model was relatively low ($R^{2}$ = 0.12). When incorporating THSA and RHSA values predicted by NetSurfP-2, the performance improved ($R^{2}$ = 0.43).

**Figure 2.**
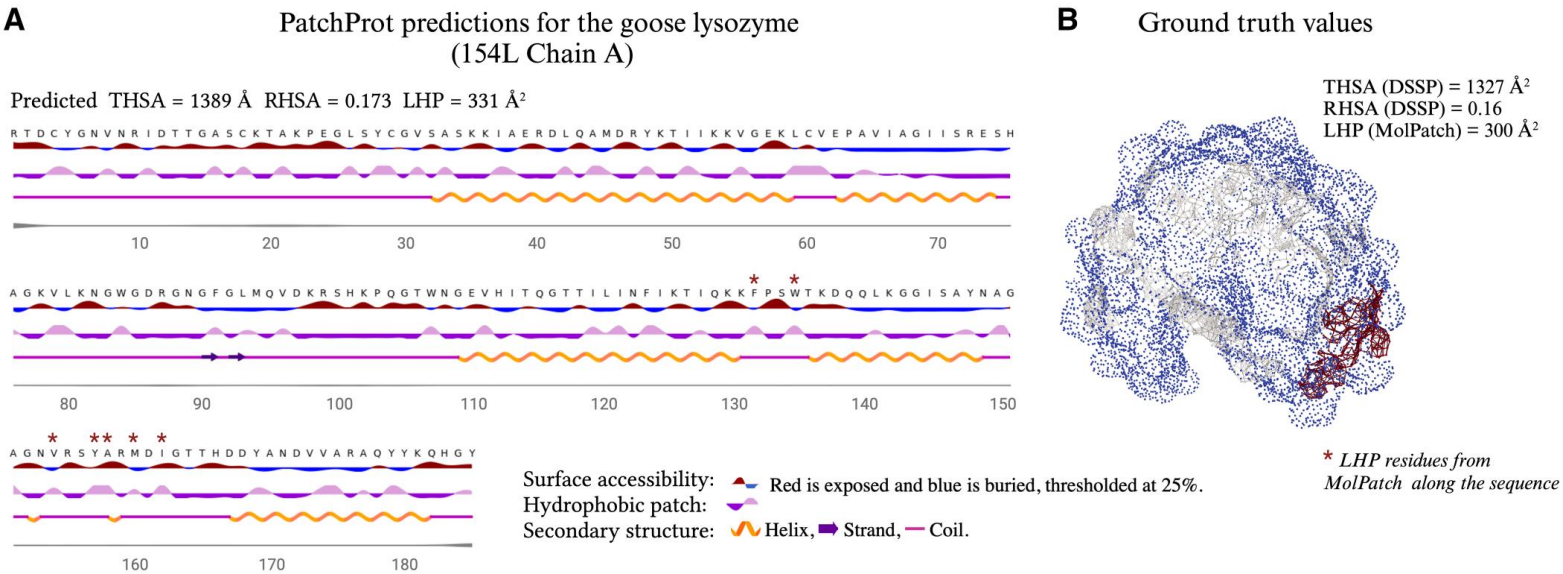
Assessment of hydrophobic patch (HP) predictions. (A) 154 L chain A—Case example from the test set of CB513. A visualization for PatchProt predictions in a manner of NetSurfP-3. (B) Ground truth labels for the same protein structure were calculated from DSSP (for THSA and RHSA and MolPatch (for the largest HP).

To assess the performance of PatchProt on global predictions for LHP values, we benchmarked our predictions against other Random Forest and XGBoost regressor models reported previously (see online supplementary material for a colour version of this figure, Fig. S2) (van Gils *et al.* 2022) (see Methods). For difficult regression tasks, $R^{2}$ or the mean absolute error (MAE) values are heavily influenced by outliers and generally do not produce results that are easy to interpret. In addition to the $R^{2}$ and MAE metrics, we evaluated the performance of the prediction model by examining the relative error threshold curve given a certain threshold, inspired by the GDT-TS score (Zemla *et al.* 2001, van Gils *et al.* 2022). See online supplementary material for a colour version of this figure, Fig. S2 shows that we not only achieved significantly higher performance at the global level ($R^{2}$ = 0.54) but also added residue-based predictions that to the best of our knowledge, have not been attempted before (Table 3). Moreover, it is possible to visualize the hydrophobic patches at a residue level in a similar manner as implemented for NetsurfP-3 (Fig. 2A) (Høie *et al.* 2022). A case example was randomly selected from the test set of CB513 and the predictions by PatchProt are comparable to the ground truth LHP area calculated by MolPatch (Fig. 2).

**Table 3.** Model performance for additional local (l) and global (g) tasks.

Test dataset	PatchProt	LHP g ↓	HP l ↑	HP l ↓	LHP l ↑	LHP l ↓	SP ↑	NX ↑
	Prediction tasks	(MAE)	(MCC)	(FNR)	(MCC)	(FNR)	(ACC)	(ACC)
CASP12	(L)HP only	588.9	0.854	0.070	0.405	0.682	–	–
	SSE, (L)HP	497.0	0.855	**0.047**	0.397	**0.619**	–	–
	SSE, (L)HP, SP, NX	**449.8**	**0.858**	0.053	**0.461**	0.706	1	1
CB513	(L)HP only	434.1	0.861	0.072	0.369	0.681	–	–
	SSE, (L)HP	418.7	**0.865**	**0.048**	**0.392**	**0.630**	–	–
	SSE, (L)HP, SP, NX	**416.7**	0.864	0.059	0.335	0.729	0.683	0.269
TS115	(L)HP only	**483.8**	0.866	0.063	0.375	0.685	–	–
	SSE, (L)HP	503.8	0.869	**0.045**	**0.419**	**0.603**	–	–
	SSE, (L)HP, SP, NX	517.8	**0.870**	0.054	0.342	0.726	0.745	0.857

Performance of our multitask model on the CB513, TS115, and CASP12 datasets compared with the multitask and single-task models. Each column reports an output variable with the corresponding metrics: Accuracy (ACC), Matthews correlation coefficient (MCC), false negative rate (FNR), and global MAE. SSE, Secondary structure element, primary tasks (Table 2), (L)HP, (largest) hydrophobic patch, NX, normalized expression, SP, species. Up- and down-facing arrows indicate metrics for which an improvement represents larger or lower values. For each dataset and prediction task, the values corresponding to the best performance are shown in bold.

To evaluate the model performance on auxiliary tasks, we compare three models: (i) The *(L)HP* only model, which is trained on predicting hydrophobic patches (both global and local) without any additional features. (ii) The SSE + (L)HP model and (iii) the final model that includes all the implemented tasks to explore whether adding less relevant global tasks would improve or worsen the performance. The *(L)HP only* model performed significantly worse than every other model. When we combine (L)HP tasks with the primary secondary structure properties, the (L)HP predictions improved suggesting the benefits of a multi-task learning strategy. Adding normalized expression values, and species improved the global LHP predictions, however, we did not observe a significant added benefit of global tasks in the residue-level performance measures.

## 4 Discussion and conclusions

In this article, we present an approach to fine-tune large language models for multi-task protein property prediction. Our method outperformed currently published best-performing models in well-established secondary structure component prediction tasks without a time-consuming multiple sequence alignment step (Table 2). An exhaustive search of homologs is a rate-limiting step for methods based on multiple sequence alignments and it can now be partially captured by protein language models leading to a substantial acceleration of predictions (Camacho *et al.* 2009, Remmert *et al.* 2011, Potter *et al.* 2018, Heinzinger *et al.* 2019, Mirdita *et al.* 2019). We believe that our improvement is possible by the pretrained ESM2 model used to encode the language of proteins, a recently published protein language model by Meta which outperforms every other model on a wide variety of tasks. By solely changing ESM-1b to ESM-2, we observe an increase in overall performance (Table 2). Even without the downstream architecture of NetSurfP-3 (convolutional encoder and bi-directional LSTM), PatchProt outperforms both NetSurfP-2 and -3 in most tasks (Table 2).

In addition to the local residue-based tasks, our model—PatchProt can predict global tasks. With this approach, we can combine relevant local and global tasks and significantly improve global predictions that are challenging otherwise (Capel *et al.* 2022). We have shown that learning a shared representation on SSE tasks improves the model’s performance across all (L)HP metrics, except for LHP global on the TS115 test set when compared to a model trained solely on hydrophobic patches (Table 3). Global auxiliary tasks, such as species and expression, appear to enhance the global LHP values, but the improvement is not consistently observed in the local (L)HP predictions (Table 3). Additionally, we have shown that adding less relevant tasks does not harm the model performance in primary prediction tasks and can even improve the performance in certain cases (Table 2). Importantly, global LHP predictions were previously demonstrated to be challenging (van Gils *et al.* 2022) and local LHP predictions, to the best of our knowledge, have never been attempted. Additionally, we obtain better predictions on added tasks through multi-tasking compared to single-task models demonstrated by hydrophobic patches. Often, biologically relevant predictions suffer from low-quality or less standardized datasets. Here we have demonstrated, that data scarcity could be tackled by combining existing datasets with limited annotations to benefit from commonalities among the prediction tasks.

Further research could usefully explore the model interpretability by separately incorporating or removing primary tasks. Moreover, other relevant residue- and protein-level properties could be explored, including solubility, aggregation propensity or post-translational modifications. One possible way to improve global predictions would be to investigate sequence-length normalization. Based on our current approach, PatchProt makes predictions for all residues, which are summed to provide a global prediction value or a class. An alternative approach to avoid potential sequence length dependencies is to use the mean of these values instead of the sum.

AlphaFold has greatly advanced our potential to utilize deep-learning methods for predicting protein structures from sequences (Jumper *et al.* 2021) allowing open access to over 200 million protein structure predictions (Varadi *et al.* 2022). Robust protein structure prediction tools allowed us to use structure-based methods to calculate protein properties, instead of predicting properties directly from an amino acid sequence (Badaczewska-Dawid *et al.* 2022). Nevertheless, structure-based calculations on predicted structures are challenging to validate. One of the challenges in calculating LHP using MolPatch from predicted AlphaFold structures arises from disordered regions, which are often excluded from experimentally defined PDB structures. Having larger surface accessibility in coiled regions or non-globular proteins, in general, can lead to overestimated LHP area calculations. To tackle this challenge, MolPatch or other structure-based tools need to be modified for predicted protein structures to exclude disordered regions.

Predicting protein properties directly from amino acid sequences is a valuable way to quickly and accurately annotate proteins. Moreover, here we focus on predicting LHPs without the need for a multiple sequence alignment. While protein foundation models offer an outstanding opportunity to improve predictions on various challenging tasks, the memory requirements of large language models can be a significant limitation for their use on resource-constrained devices. In addition to the state-of-the-art methods, [e.g. pruning and knowledge distillation (Hong *et al.* 2022, Xu *et al.* 2022)], quantization schemes can be explored to reduce the memory footprint of large language models by representing the model parameters using fewer bits (Dettmers *et al.* 2023, 2024, Xiao *et al.* 2023).

To summarize, with our model architecture that combines an advanced fine-tuning strategy with related task prediction, we not only demonstrate the possibility to outperform state-of-the-art tools in established secondary structure element predictions but also to add prediction tasks that are challenged with data scarcity or intrinsic difficulty. Specifically, our analysis shows that including related residue-level tasks can improve performance on more difficult global tasks, such as LHP areas. Our approach can be applied to other complex global properties, such as turnover, aggregation-propensity, and solubility prediction tasks. Continued research in large language models will further enhance the effectiveness and applicability of fine-tuning and leveraging powerful representations of protein sequences for various relevant tasks.

## Supplementary Material

vbae154_Supplementary_Data

## Data Availability

Data and code implemented in this study are available at: https://github.com/Deagogishvili/chapter-multi-task
