# Supplementary material for: PatchProt: hydrophobic patch prediction using protein foundation models
Source: Bioinform Adv. 2024 Oct 14;4(1):vbae154. doi: 10.1093/bioadv/vbae154 (PMC11525051; doi:10.1093/bioadv/vbae154)
Supplement: vbae154_Supplementary_Data [file vbae154_supplementary_data.pdf]

Supplementary information

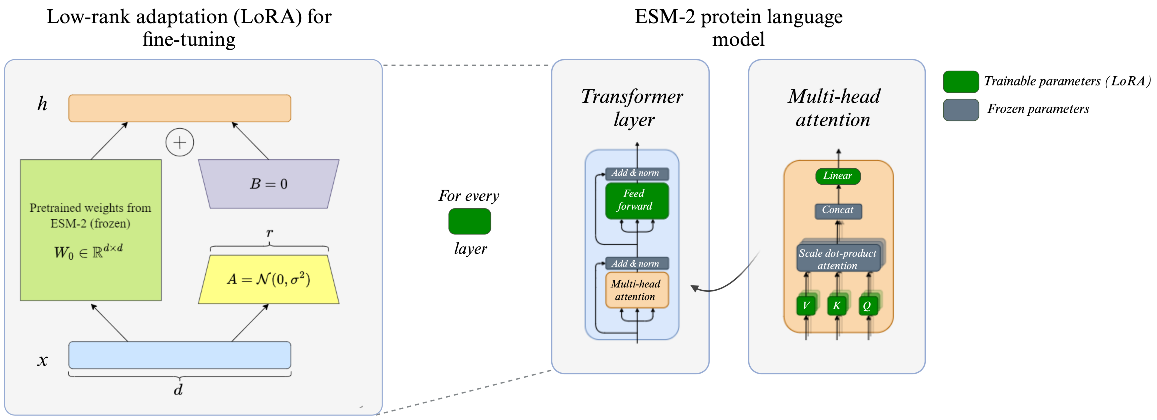

Figure S1. Fine-tuning strategy with Low-Rank Adaptation (LoRA). To efficiently fine-tune the foundation model, we adopted recent advancements in parameter-efficient fine-tuning LoRA. In our approach, we applied LoRA to every linear layer within the original transformer architecture [33], significantly reducing the number of updated parameters (to $2rd$ from the layer's original d^2). W_0 denotes the original weight matrix, decomposed into two lower-rank matrices, A and B, with dimensions $r \times d$ and $d \times r$ respectively.

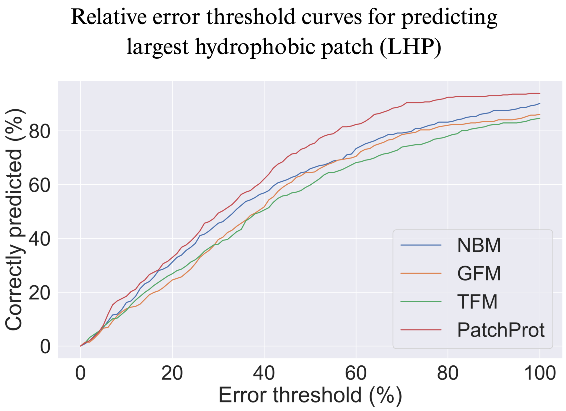

Figure S2. Benchmarking global largest hydrophobic predictions (LHP). The Accuracy of the predictions of the largest patch hydrophobic surface area was compared using threshold curves. Global predictions by PatchProt are benchmarked against other methods, including the three-feature model (TFM), which uses the sequence length, number of hydrophobic amino acids and number of hydrophilic amino acids as input features [45]. The global feature model (GFM) trained on 31 global features using an XGBoost regressor [46]. NetSurfP-2.0-based model (NBM), which is a random forest model trained using the relative and total hydrophobic surface area values (THSA, RHSA) predicted by NetSurfP-2.0, since the LHP cannot be calculated from NetSurfP-2.0 predictions directly [1]. The fraction of correctly predicted proteins within a certain error margin for each of the methods is shown as calculated over the test set. The test set and the threshold curve calculations were replicated from the previous study [1]. Importantly, the large fraction of proteins in the test set were used to train PatchProt. For a fair comparison, the overlapping proteins were removed and the curves were calculated for the rest of the proteins in the test set (n=346).
